# Association between Mediterranean diet adherence and dyspeptic symptoms in older adults: a cross-sectional study in a geriatric outpatient population

**DOI:** 10.1186/s12877-026-07501-y

**Published:** 2026-04-17

**Authors:** Nezihe Otay Lule, Kemal Ozan Lule, Zeynel Abidin Ozturk

**Affiliations:** 1 Department of Nutrition and Dietetics, Faculty of Health Sciences, Gaziantep University, Gaziantep, Türkiye; 2 Department of Internal Medicine, Faculty of Medicine, Gaziantep University, Gaziantep, Türkiye; 3 Department of Internal Medicine, Faculty of Medicine, Division of Geriatrics, Gaziantep University, Gaziantep, Türkiye

**Keywords:** Geriatrics, Dyspepsia, Mediterranean diet, Severity of Dyspepsia Assessment (SODA), MEDAS

## Abstract

**Background:**

Dyspeptic symptoms are common in older adults and may impair nutritional status and quality of life. Dietary patterns are increasingly recognized as modifiable determinants of gastrointestinal symptom burden, yet evidence regarding Mediterranean diet adherence and dyspepsia severity in geriatric populations remains limited. We aimed to examine the association between Mediterranean diet adherence and dyspeptic symptom severity—including pain, non-pain symptoms, and symptom-related satisfaction—among adults aged ≥ 65 years presenting with dyspeptic complaints.

**Methods:**

This cross-sectional study was conducted between January 2023 and December 2024 in a geriatric outpatient clinic at Gaziantep University Training and Research Hospital. Consecutive adults aged ≥ 65 years with dyspeptic symptoms lasting ≥ 4 weeks and adequate cognitive capacity (clinical judgment and/or MMSE ≥ 24) were enrolled. Mediterranean diet adherence was assessed using the 14-item Mediterranean Diet Adherence Screener (MEDAS; score 0–14) and categorized as low (≤ 5), moderate (6–9), or high (≥ 10). Dyspeptic symptoms were evaluated using the interviewer-administered Severity of Dyspepsia Assessment (SODA) scale (pain, non-pain symptoms, satisfaction; 7-day recall). Pearson correlations, one-way ANOVA with appropriate post-hoc tests, and multivariable linear regression analyses were performed; models were adjusted for age, sex, and BMI as clinically relevant covariates.

**Results:**

A total of 165 participants were included (70.9% women; mean age 71.47 ± 6.43 years; mean BMI 28.51 ± 4.99 kg/m^2^). Most exhibited moderate MEDAS adherence (73.9%), while 18.2% had low and 7.9% high adherence. Higher MEDAS scores were associated with lower SODA pain (*r =* − 0.177, *p =* 0.02) and non-pain symptom scores (*r =* − 0.429, *p <* 0.001), and higher satisfaction scores (indicating better perceived gastrointestinal well-being; *r =* 0.431, *p <* 0.001). SODA subscale scores differed significantly across adherence categories (all *p <* 0.05), whereas BMI did not (*p =* 0.521). In adjusted regression models, each one-point increase in MEDAS was associated with lower pain (B = − 1.50, *p =* 0.001), non-pain symptoms (B = − 1.66, *p <* 0.001), and higher satisfaction (B = 0.77, *p <* 0.001).

**Conclusions:**

Greater Mediterranean diet adherence was independently associated with lower dyspeptic symptom severity and higher symptom-related satisfaction in older adults with dyspepsia. Prospective and interventional studies are warranted to determine whether improving Mediterranean diet adherence can causally reduce dyspeptic symptoms in geriatric care.

**Supplementary Information:**

The online version contains supplementary material available at 10.1186/s12877-026-07501-y.

## Background

Dyspeptic symptoms, including epigastric pain, early satiety, postprandial fullness, and bloating, are frequently reported in older adults and constitute a substantial burden on geriatric healthcare [1]. The prevalence of dyspepsia in the elderly has been estimated to range between 20 and 40%, depending on diagnostic criteria and clinical setting, with symptoms often being more persistent and debilitating than in younger populations [2]. As global life expectancy increases, dyspeptic complaints are expected to contribute increasingly to reduced quality of life, impaired nutritional status, and higher healthcare utilization among older individuals.

Age-related physiological changes in the gastrointestinal (GI) system play a central role in this increased symptom burden. Delayed gastric emptying, altered gastrointestinal motility, reduced splanchnic blood flow, diminished visceral sensitivity thresholds, and age-associated changes in gut microbiota composition have all been implicated in the heightened susceptibility to dyspeptic symptoms observed in geriatric populations [3]. In addition, multimorbidity, polypharmacy, and the frequent use of gastroduodenally active medications may further exacerbate or mimic dyspeptic manifestations in older adults.

Dietary patterns are increasingly recognized as modifiable determinants of gastrointestinal symptomatology [4]. The Mediterranean diet—characterized by high consumption of fruits, vegetables, legumes, whole grains, nuts, and olive oil; moderate intake of fish and dairy products; and limited consumption of red meat and processed foods—has been consistently associated with favorable metabolic, cardiovascular, and gastrointestinal outcomes [5]. Its anti-inflammatory properties, lower saturated fat content, and beneficial effects on gut motility and microbiota composition suggest a potential protective role against functional gastrointestinal disorders, including dyspepsia [6]. Although emerging evidence indicates that greater adherence to the Mediterranean diet may be associated with reduced prevalence or severity of upper GI symptoms, existing findings remain inconsistent and are largely derived from non-geriatric populations [7, 8].

Despite growing interest in dietary strategies for functional gastrointestinal disorders, data specifically addressing the relationship between Mediterranean diet adherence and dyspeptic symptom severity in older adults remain scarce. This represents a critical knowledge gap, given that older individuals are uniquely vulnerable to both nutritional imbalance and gastrointestinal dysfunction, and that non-pharmacological approaches are particularly attractive in the context of polypharmacy and multimorbidity. Notably, no previous study has evaluated this association using both the validated 14-item Mediterranean Diet Adherence Screener (MEDAS) and a multidimensional dyspepsia assessment instrument such as the Severity of Dyspepsia Assessment (SODA). Therefore, the present study aimed to examine the association between Mediterranean diet adherence and dyspeptic symptom severity—including pain, non-pain gastrointestinal symptoms, and symptom-related satisfaction—among adults aged 65 years and older presenting with dyspeptic complaints to a geriatric outpatient clinic. Clarifying this relationship may help inform dietary recommendations and support the development of non-pharmacological strategies for dyspepsia management in geriatric care.

## Methods

**Fig. 1:**
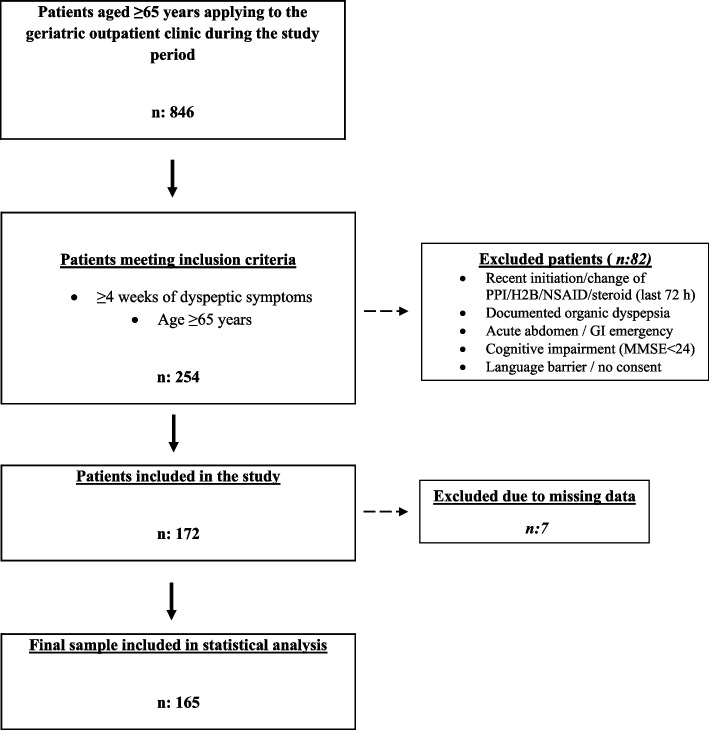
Study flow chart. A total of 846 patients aged ≥65 years were screened for eligibility. After applying inclusion and exclusion criteria, 254 patients met eligibility requirements; 82 were excluded due to recent initiation or change of PPI/H2-blocker/NSAID/steroid therapy, documented organic dyspepsia, acute abdomen or gastrointestinal emergency, cognitive impairment (MMSE <24), or language barrier/lack of consent. Of the 172 patients initially enrolled, 7 were subsequently excluded due to missing data, yielding a final analytical sample of 165 participants. Abbreviations: GI, gastrointestinal; MMSE, Mini-Mental State Examination; NSAID, nonsteroidal anti-inflammatory drug; PPI, proton pump inhibitor

This cross-sectional study was conducted between January 2023 and December 2024 at the Department of Internal Medicine, Division of Geriatrics, Gaziantep University Training and Research Hospital. The study population consisted of adults aged ≥ 65 years who presented to the geriatric outpatient clinic during the study period. Consecutive patients were screened for eligibility to minimize selection bias. Individuals with upper gastrointestinal symptoms consistent with dyspepsia lasting for ≥ 4 weeks, adequate cognitive capacity to complete the questionnaires, and willingness to participate were enrolled in the study(Fig. 1).

### Sample size estimation

Sample size was determined based on the primary aim of assessing the correlation between the Mediterranean Diet Adherence Screener (MEDAS) and the subscales of the Severity of Dyspepsia Assessment (SODA). Assuming a weak correlation (*r =* 0.30), as reported in previous literature [8], an a priori power analysis (G*Power 3.1.9; two-tailed, α = 0.05, 1–β = 0.80) indicated a minimum required sample size of 84 participants.

### Inclusion criteria

- Age ≥ 65 years
- Presentation to the geriatric outpatient clinic
- Presence of dyspeptic symptoms lasting ≥ 4 weeks, including epigastric pain or burning, early satiety, postprandial fullness, or bloating
- Adequate cognitive capacity to complete the study questionnaires, as determined by clinical judgment and/or a Mini-Mental State Examination (MMSE) score ≥ 24
- Ability and willingness to provide written informed consent and participate voluntarily in the study

### Exclusion criteria

- Recent initiation or dose modification (within the past 72 h) of proton pump inhibitors, H2-receptor blockers, nonsteroidal anti-inflammatory drugs, or systemic corticosteroids
- Acute abdominal pain, gastrointestinal emergencies, or suspected surgical abdomen
- Documented organic causes of dyspepsia, including peptic ulcer disease, gastrointestinal malignancy, complicated gastroesophageal reflux disease, or pancreatitis
- Cognitive impairment precluding valid questionnaire completion (MMSE < 24)
- Language or communication barriers preventing reliable data collection
- Refusal to participate or inability to provide written informed consent

Organic causes of dyspepsia were excluded on the basis of available medical records, including prior endoscopic and imaging findings documented in the hospital information system. However, systematic endoscopic evaluation was not performed as part of the present study, and formal diagnostic criteria for functional dyspepsia (e.g., Rome IV) were not applied. Accordingly, the study population comprises older adults presenting with dyspeptic symptoms of at least four weeks' duration in whom documented organic pathology had been ruled out, rather than a formally diagnosed functional dyspepsia cohort. Helicobacter pylori infection status was not assessed. Similarly, gastroparesis was not systematically screened.

The study was conducted in line with the ethical principles outlined in the Declaration of Helsinki. The study was approved by Gaziantep University Non-Interventional Clinical Research Ethics Committee (Approval date/number: 2021/101). The study followed the STROBE reporting guidelines.

### Data collection

Data were collected through structured, interviewer-administered face-to-face interviews. The questionnaire captured sociodemographic characteristics (age, sex, marital status, education level, and perceived income status), anthropometric measurements (body weight and height), physician-diagnosed chronic conditions, medication use (particularly proton pump inhibitors, H2-receptor blockers, and nonsteroidal anti-inflammatory drugs), and dietary habits, including the number of daily meals and snacks and meal-skipping behaviors (Supplementary File 1).

Adherence to the Mediterranean diet was assessed using the validated 14-item Mediterranean Diet Adherence Screener (MEDAS). Dyspeptic symptoms were evaluated using the Severity of Dyspepsia Assessment (SODA) scale, which quantifies symptom severity over the preceding 7 days.

### Mediterranean Diet Adherence Screener (MEDAS)

The validated 14-item Mediterranean Diet Adherence Screener (MEDAS) consists of 12 items assessing food consumption frequency and 2 items evaluating dietary behaviors. Each item is scored as 0 or 1, yielding a total score ranging from 0 to 14. In accordance with established cut-off values commonly used in Mediterranean diet research, adherence levels were categorized as low (≤ 5 points), moderate (6–9 points), and high (≥ 10 points) [9]. The Turkish validation study demonstrated strong reliability and construct validity in the adult population [10]. Notably, the Turkish validation sample did not specifically include adults aged 65 years or older, which represents a potential limitation of instrument validity in the present study population. To partially mitigate comprehension and recall difficulties among geriatric participants, the questionnaire was administered in an interviewer-assisted format.

### Severity of Dyspepsia Assessment (SODA) interviewer-administered

The Severity of Dyspepsia Assessment (SODA) is a validated instrument designed to assess dyspeptic symptom severity over the preceding 7 days. Although originally developed as a self-reported measure, the scale was administered in an interviewer-assisted format in the present study to minimize potential recall bias and comprehension difficulties among older adults.

The SODA comprises three subscales: Pain Intensity (6 items; score range 2–47), Non-pain Symptoms (7 items; score range 7–35), and Satisfaction (4 items; score range 2–23). Higher scores on the pain and non-pain symptom subscales indicate greater symptom severity, whereas higher scores on the satisfaction subscale reflect better perceived gastrointestinal well-being [11]. The Turkish validity and reliability study of SODA was completed in 2006 [12]. The scale was not used for diagnostic purposes but solely to assess the severity of dyspeptic symptoms and patient satisfaction.

Permission to use the MEDAS and the SODA scale was obtained via e-mail from the authors who conducted the Turkish validity and reliability studies of these instruments prior to data collection.

### Statistical analysis

Statistical analyses were performed using the Statistical Package for the Social Sciences (SPSS), version 22.0 (IBM Corp., Armonk, NY, USA). Categorical variables were summarized as counts (n) and percentages (%), while continuous variables were presented as mean ± standard deviation (SD) together with minimum and maximum values.

The distribution of continuous variables was assessed using the Shapiro–Wilk test and visual inspection of histograms and normal probability plots. As the variables demonstrated approximately normal distributions, parametric statistical tests were applied. Comparisons between two independent groups were conducted using the independent samples t-test, while comparisons among more than two groups were performed using one-way analysis of variance (ANOVA). Homogeneity of variances was evaluated using Levene’s test.

Following significant overall ANOVA results, post-hoc pairwise comparisons were performed using the Games–Howell test for all variables. Post-hoc analyses were not conducted when the overall ANOVA was non-significant.

Associations between MEDAS scores and SODA subscale scores were examined using Pearson correlation analysis. Multivariable linear regression analyses were subsequently conducted to assess independent associations between Mediterranean diet adherence and SODA pain, non-pain symptom, and satisfaction scores. Age, sex, and body mass index (BMI) were included as covariates based on clinical relevance. All regression models were constructed using the enter method. Because the high-adherence group was relatively small, sensitivity analyses were performed by repeating the multivariable linear regression models after excluding participants in this group, in order to examine the robustness of the associations between MEDAS score and SODA subscale scores.

Multicollinearity was assessed using variance inflation factors (VIF), with all VIF values below 2, indicating no significant multicollinearity. Model explanatory power was evaluated using R^2^ and adjusted R^2^ values. Regression assumptions were assessed through residual diagnostics, including evaluation of residual normality (histograms and normal P–P plots) and homoscedasticity (scatter plots of standardized residuals versus predicted values). Given the cross-sectional study design, Durbin–Watson statistics were reported for completeness but were not interpreted as indicators of autocorrelation. A two-sided p value < 0.05 was considered statistically significant for all analyses.

## Results

### Baseline characteristics

Table 1 summarizes the sociodemographic and clinical characteristics of the study population. A total of 165 older adults were included in the analysis. The cohort consisted predominantly of women (70.9%) and was characterized by a high prevalence of cardiometabolic comorbidities, including cardiovascular diseases (63.6%) and diabetes mellitus (60.0%). Most participants exhibited moderate adherence to the Mediterranean diet (73.9%), while 18.2% and 7.9% demonstrated low and high adherence, respectively. The mean age of the participants was 71.47 ± 6.43 years, and the mean body mass index (BMI) was 28.51 ± 4.99 kg/m^2^. Mean MEDAS score was 6.85 ± 1.68, indicating overall moderate dietary adherence. Mean SODA scores were 17.83 ± 10.44 for pain, 17.10 ± 5.79 for non-pain symptoms, and 10.96 ± 3.20 for satisfaction.

**Table 1:** Sociodemographic and clinical characteristics of the study population (*n =* 165)

Variable	**Value**
Sociodemographic characteristics *n* (%)	
Sex, Female	117 (70.9)
Sex, Male	48 (29.1)
Marital status, Married	108 (65.5)
Marital status, Single	57 (34.5)
No formal education	27 (16.4)
Primary school	123 (74.5)
Middle school—high school	15 (9.1)
Living alone	33 (20.0)
Living with family	132 (80.0)
Socioeconomic status *n* (%)	
Income higher than expenses	27 (16.4)
Income lower than expenses	69 (41.8)
Income equal to expenses	69 (41.8)
Clinical characteristics (physician-diagnosed)* *n* (%)	
No comorbidity	3 (1.8)
Cardiovascular comorbidities	105 (63.6)
Diabetes mellitus	99 (60.0)
Other conditions**	30 (18.1)
Mediterranean diet adherence (MEDAS) category *n* (%)	
Low adherence	30 (18.2)
Moderate adherence	122 (73.9)
High adherence	13 (7.9)
BMI category *n* (%)	
Underweight	3 (1.8)
Normal weight	48 (29.1)
Overweight	48 (29.1)
Obese	66 (40.0)
Continuous variables (mean ± SD (min–max)	
Age, years	71.47 ± 6.43 (65–89)
BMI, kg/m^2^	28.51 ± 4.99 (18.42–42.97)
MEDAS score	6.85 ± 1.68 (3–11)
SODA Pain score	17.83 ± 10.44 (2–47)
SODA Non-pain score	17.10 ± 5.79 (7–35)
SODA Satisfaction score	10.96 ± 3.20 (2–22)

Values are presented as mean ± standard deviation (minimum–maximum) or number (percentage), as appropriate

*BMI* Body mass index, *MEDAS* Mediterranean Diet Adherence Screener, *SODA* Severity of Dyspepsia Assessment

^*^Multiple responses were permitted

^**^Asthma/COPD, Cancer, Osteoporosis, hypothyroidism, depression, chronic kidney failure

Sociodemographic and lifestyle factors associated with Mediterranean diet adherence categories are presented in Supplementary Table S1.

### Correlation analyses

Pearson correlation analyses demonstrated significant associations between Mediterranean diet adherence and all SODA subscale scores (Table 2). Higher MEDAS scores were weakly but significantly associated with lower pain intensity scores (*r =* − 0.177, *p =* 0.023) and moderately associated with lower non-pain symptom scores (*r =* − 0.429, *p <* 0.001). In contrast, MEDAS scores showed a moderate positive correlation with SODA satisfaction scores (*r =* 0.431, *p <* 0.001), indicating greater symptom-related satisfaction with increasing adherence to the Mediterranean diet.

**Table 2:** Correlations between Mediterranean diet adherence and SODA subscale scores

Variable	MEDAS score	SODA Pain	SODA Non-pain	SODA Satisfaction
MEDAS score	1	-	-	-
SODA Pain	− 0.177*	1	-	-
SODA Non-pain	− 0.429**	0.453**	1	-
SODA Satisfaction	0.431**	− 0.236**	− 0.382**	1

Pearson correlation coefficients are shown

^*^p < 0.05, **p < 0.001

### Group comparisons according to Mediterranean diet adherence

Given the observed linear associations, group-based comparisons were conducted according to Mediterranean diet adherence categories (Table 3). BMI did not differ significantly across adherence groups (F = 0.66, *p =* 0.521).

**Table 3:** Comparison of BMI and SODA subscale scores across Mediterranean diet adherence groups

Variable	Low adherence (*n =* 30)	Moderate adherence (*n =* 122)	High adherence (*n =* 13)	F	*p*-value
BMI (kg/m^2^)	29.21 ± 6.11	28.46 ± 4.77	27.34 ± 4.15	0.66	0.521
SODA Pain score	18.33 ± 8.91^a^	18.62 ± 10.85^a^	9.23 ± 4.99^b^	5.03	**0.008**
SODA Non-pain score	22.67 ± 6.48^a^	16.11 ± 4.88^b^	13.54 ± 3.89^b^	22.97	**< 0.001**
SODA Satisfaction score	8.03 ± 3.59^a^	11.42 ± 2.66^b^	13.46 ± 2.67^c^	22.40	**< 0.001**

Values are presented as mean ± standard deviation. Group comparisons were performed using one-way analysis of variance (ANOVA). Post-hoc pairwise comparisons were conducted using the Games–Howell test for all variables. Different superscript letters indicate statistically significant differences between groups (*p <* 0.05)

Bold p-values indicate statistical significance (*p* < 0.05)

In contrast, significant differences were observed across all SODA subscales. Pain intensity scores differed significantly between adherence groups (F = 5.03, *p =* 0.008). Post-hoc Games–Howell analyses revealed that participants with high Mediterranean diet adherence had significantly lower pain scores compared with both low (*p =* 0.022) and moderate adherence groups (*p =* 0.005), while no significant difference was observed between low and moderate adherence groups. However, given the small sample size of the high-adherence group (*n =* 13), these pairwise comparisons should be interpreted with caution, as the group means may be sensitive to individual outliers and the associated confidence intervals are expected to be wide.

Similarly, non-pain symptom scores varied markedly across adherence categories (F = 22.97, *p <* 0.001). Post-hoc analyses showed that participants with low adherence exhibited significantly higher non-pain symptom scores compared with both moderate and high adherence groups (both *p <* 0.001), whereas no significant difference was observed between moderate and high adherence groups.

Significant differences were also observed for SODA satisfaction scores (F = 22.40, *p <* 0.001). Post-hoc Games–Howell analyses showed that satisfaction scores increased progressively across low, moderate, and high adherence groups, with all pairwise comparisons reaching statistical significance (low vs. moderate: *p <* 0.001; low vs. high: *p <* 0.001; moderate vs. high: *p =* 0.048).

### Multivariable regression analyses

In multivariable linear regression analysis, higher Mediterranean diet adherence was independently associated with lower pain intensity scores (Table 4). After adjustment for age, sex, and BMI, each one-point increase in MEDAS score was associated with a 1.5-point decrease in SODA pain intensity (β = − 0.242, *p =* 0.001). Female sex remained a strong independent predictor of higher pain intensity scores (β = 0.373, *p <* 0.001), whereas BMI was not significantly associated with pain intensity.

**Table 4:** Multivariable linear regression analysis of factors associated with SODA pain intensity

Variable	B (95% CI)	Standardized β	*p* value
Age (years)	0.25 (0.01 to 0.49)	0.157	**0.038**
Sex (female vs male)	8.55 (5.02 to 12.07)	0.373	**< 0.001**
BMI (kg/m^2^)	− 0.03 (− 0.35 to 0.29)	− 0.014	0.853
MEDAS score	− 1.50 (− 2.42 to − 0.59)	− 0.242	**0.001**
*Model statistics: R*^*2*^ = *0.162, Adjusted R*^*2*^ = *0.141, F* = *7.742, p <* *0.001, Durbin–Watson ≈ 1.53*			

The model was adjusted for age, sex, body mass index (BMI), and Mediterranean Diet Adherence Score (MEDAS)

No multicollinearity was detected (all VIF values < 1.2.)

Bold p-values indicate statistical significance (*p* < 0.05)

Higher Mediterranean diet adherence was also independently associated with lower non-pain symptom scores (Table 5). Each one-point increase in MEDAS score corresponded to a 1.66-point reduction in non-pain symptom severity (β = − 0.481, *p <* 0.001). Female sex remained an independent predictor of higher non-pain symptom scores, while age was not significantly associated. This model explained 24.7% of the variance in non-pain symptom scores.

**Table 5:** Multivariable linear regression analysis of factors associated with SODA non-pain symptom scores

Variable	B (95% CI)	Standardized β	*p* value
Age (years)	0.08 (− 0.05 to 0.20)	0.087	0.219
Sex (female vs male)	3.83 (2.00 to 5.66)	0.301	**< 0.001**
BMI (kg/m^2^)	− 0.03 (− 0.19 to 0.13)	− 0.025	0.722
MEDAS score	− 1.66 (− 2.14 to − 1.18)	− 0.481	**< 0.001**
Model statistics: *R*^2^ = 0.265, Adjusted *R*^2^ = 0.247, *F* = 14.42, *p <* 0.001, Durbin–Watson ≈ 1.32			

The model was adjusted for age, sex, body mass index (BMI) and Mediterranean Diet Adherence Score (MEDAS)

No multicollinearity was detected (all VIF values < 1.2.)

Bold p-values indicate statistical significance (*p* < 0.05)

In the fully adjusted model for SODA satisfaction (Table 6), Mediterranean diet adherence was independently associated with higher satisfaction scores (β = 0.403, *p <* 0.001). In contrast, BMI was inversely associated with satisfaction (β = − 0.195, *p =* 0.008), while age and sex were not significantly associated with satisfaction scores.

**Table 6:** Multivariable linear regression analysis of factors associated with SODA satisfaction score

Variable	B (95% CI)	Standardized β	*p* value
Age (years)	0.01 (− 0.06 to 0.08)	0.027	0.704
Sex (female vs male)	0.03 (− 1.01 to 1.07)	0.004	0.959
BMI (kg/m^2^)	− 0.13 (− 0.22 to − 0.03)	− 0.195	**0.008**
MEDAS score	0.77 (0.50 to 1.04)	0.403	**< 0.001**
Model statistics:*R*^2^ = 0.225, Adjusted *R*^2^ = 0.205, *F* = 11.60, *p <* 0.001, Durbin–Watso*n =* ≈ 1.01			

The model was adjusted for age, sex, body mass index (BMI), and Mediterranean Diet Adherence Score (MEDAS)

No multicollinearity was detected (all VIF values < 1.2.)

The relatively low Durbin–Watson value (≈1.01) may reflect residual clustering from unmeasured variables or sequential enrollment of participants with similar clinical profiles rather than true serial dependence, consistent with the cross-sectional design

Bold p-values indicate statistical significance (*p* < 0.05)

In sensitivity analyses excluding participants in the high-adherence group, the association between MEDAS score and pain intensity was attenuated and no longer statistically significant after adjustment for age, sex, and BMI (B = − 0.63, 95% CI: − 1.76 to 0.50; standardized β = − 0.084; *p =* 0.275). In contrast, higher MEDAS score remained independently associated with lower non-pain symptom scores (B = − 1.87, 95% CI: − 2.46 to − 1.28; standardized β = − 0.450; *p <* 0.001) and higher satisfaction scores (B = 0.80, 95% CI: 0.46 to 1.14; standardized β = 0.354; *p <* 0.001). These findings suggest that the associations with non-pain symptoms and satisfaction were not driven solely by the small number of participants in the high-adherence category (Supplementary Table S2).

### Factors associated with Mediterranean diet adherence

Selected sociodemographic and lifestyle factors associated with Mediterranean diet adherence were examined using chi-square analyses (Supplementary Table S1). Mediterranean diet adherence differed significantly according to sex, marital status, and living arrangement, with higher adherence observed predominantly among women and participants living with family (all *p <* 0.05). In contrast, no significant associations were observed between Mediterranean diet adherence and BMI category, educational level, or perceived income–expense balance (all *p >* 0.05).

Meal-skipping behaviors were also significantly associated with Mediterranean diet adherence. Participants with higher adherence were more likely to never skip breakfast, dinner, and snacks (all *p <* 0.05), whereas lunch skipping was not significantly associated with adherence level (*p =* 0.255).

## Discussion

Taken together, the present findings demonstrate a consistent and graded association between Mediterranean diet adherence and dyspeptic symptom profiles in older adults. In multivariable regression models treating Mediterranean diet adherence as a continuous variable—and supported by correlation analyses and group-based comparisons—higher adherence to the Mediterranean diet was associated with lower pain and non-pain dyspeptic symptom severity and greater symptom-related satisfaction, independent of age, sex, and body mass index. Notably, these associations were strongest for non-pain symptoms and satisfaction, suggesting that dietary quality may be particularly relevant for meal-related gastrointestinal complaints and perceived well-being in geriatric populations. These results provide preliminary evidence supporting further exploration of potential mechanisms and clinical implications of Mediterranean diet adherence in the management of dyspeptic symptoms among older adults.

One of the principal findings of this study is the robust association between Mediterranean diet adherence and non-pain dyspeptic symptoms, which demonstrated both moderate correlation coefficients and the largest effect sizes in multivariable models. Non-pain symptoms such as postprandial fullness, bloating, and early satiety are particularly prevalent in older adults and are strongly influenced by gastric accommodation, gastrointestinal motility, and visceral sensitivity [3]. The Mediterranean diet, characterized by high fiber content, unsaturated fats, and antioxidant-rich foods, has been hypothesized to influence these mechanisms through improved gastric emptying dynamics, reduced low-grade inflammation, and a healthier gut microbiota composition [13]. However, the cross-sectional design of the present study does not permit determination of whether dietary quality influences symptom burden or whether individuals with more severe non-pain symptoms restrict their intake of fiber-rich, raw, or voluminous foods—thereby scoring lower on the MEDAS—as a behavioral adaptation to gastrointestinal discomfort. The magnitude and consistency of the observed association are compatible with both explanations, and prospective data are needed to distinguish between them.

Pain-related dyspeptic symptoms were also inversely associated with Mediterranean diet adherence, although the strength of this relationship was comparatively weaker. Importantly, multivariable analysis demonstrated that Mediterranean diet adherence remained an independent predictor of lower pain intensity after controlling for age, sex, and BMI. Female sex emerged as a strong independent predictor of higher pain scores, consistent with previous literature suggesting greater symptom perception, visceral hypersensitivity, [14] and healthcare-seeking behavior among women [15]. Notably, in sensitivity analyses excluding the high-adherence group, the association between MEDAS and pain intensity was attenuated and no longer statistically significant, whereas the associations with non-pain symptoms and satisfaction remained robust (Supplementary Table S2). This suggests that the pain–diet relationship in the primary analysis may have been influenced by the small number of high-adherence participants and should be interpreted with particular caution. These findings underscore the multifactorial nature of dyspeptic pain in older adults and suggest that while dietary quality is independently associated with pain severity, biological and psychosocial factors remain important determinants of pain perception.

Another notable finding is the positive association between Mediterranean diet adherence and SODA satisfaction scores. Higher satisfaction reflects better perceived gastrointestinal well-being and symptom control, which is particularly relevant in geriatric care, where quality of life and functional status are central outcomes. The independent association between higher BMI and lower satisfaction scores suggests that excess body weight may negatively influence symptom perception or treatment response, potentially through mechanisms such as increased intra-abdominal pressure, altered gastroesophageal physiology, or metabolic inflammation [16].

To contextualize the magnitude of these associations, each one-point increase in MEDAS score was associated with a 1.50-point reduction in SODA pain and a 1.66-point reduction in non-pain symptom scores. Across the observed MEDAS range in this sample (approximately 8 points), these coefficients correspond to estimated differences of approximately 12 and 13 points on the pain (range 2–47) and non-pain (range 7–35) subscales, respectively. While no established minimal clinically important difference has been defined for the SODA in geriatric populations, these magnitudes represent a substantial proportion of the observed score distributions and suggest potentially meaningful symptom relief. Nevertheless, these estimates should be interpreted cautiously given the cross-sectional design and the possibility of residual confounding.

The continuous regression analyses constitute the primary evidence for a graded association between Mediterranean diet adherence and dyspeptic symptom profiles, as they are not dependent on category-based groupings or on the small number of participants in the high-adherence group. Group-based analyses provided broadly supportive findings; however, pairwise comparisons involving the high-adherence group should be interpreted with caution given the limited sample size, which renders the group means susceptible to the influence of individual observations. Notably, BMI did not differ significantly across adherence categories, suggesting that the observed symptom differences were not merely attributable to body weight but rather to qualitative aspects of dietary intake. This finding reinforces the concept that dietary composition, rather than caloric load alone, may influence dyspeptic symptomatology [17].

From a geriatric perspective, these findings are clinically meaningful. Older adults frequently experience polypharmacy, multimorbidity, and increased susceptibility to adverse drug effects, which can complicate pharmacological management of dyspepsia [18]. Non-pharmacological interventions, including dietary modification, therefore represent an attractive and potentially safer strategy. The Mediterranean diet, already recommended for cardiovascular and metabolic health, [19, 20] may represent a promising candidate for symptom management if future interventional studies confirm a causal benefit on dyspeptic symptoms and patient satisfaction.

This study also addresses an important gap in the literature by simultaneously employing a validated dietary adherence tool (MEDAS) and a multidimensional dyspepsia assessment instrument (SODA) in an exclusively older population. Previous studies examining diet and dyspepsia have largely focused on younger or mixed-age cohorts and have often relied on symptom presence rather than severity or patient-reported satisfaction. The present findings extend existing evidence by demonstrating that Mediterranean diet adherence is associated not only with symptom severity but also with subjective gastrointestinal well-being in older adults.

### Limitations

Several limitations warrant consideration. First, and most critically, the cross-sectional design precludes causal inference. Reverse causality represents the most plausible alternative explanation: individuals with severe dyspeptic symptoms—particularly postprandial fullness, bloating, and early satiety—may restrict their intake of fiber-rich foods, raw vegetables, and large meals as a behavioral adaptation, thereby scoring lower on the MEDAS independently of any causal dietary effect. This possibility is especially pertinent in a geriatric population, and the observed associations should not be interpreted as evidence that improving dietary adherence will reduce symptom severity.

Second, the MEDAS was used rather than detailed dietary records, which may introduce recall and social desirability bias. Moreover, the Turkish validation did not include adults aged ≥ 65 years, raising an instrument validity concern: age-related factors such as appetite dysregulation, dental limitations, and food access constraints may distort MEDAS responses—for instance, low nut or raw vegetable consumption may reflect functional limitations rather than poor adherence. The interviewer-assisted format partially mitigated comprehension difficulties but cannot compensate for the lack of geriatric-specific validation.

Third, the regression models were adjusted only for age, sex, and BMI. Chronic PPI, NSAID, and opioid use were not systematically recorded in the analytical dataset and could not be included as covariates, despite their direct effects on dyspeptic symptoms and potential correlation with dietary patterns. Similarly, the absence of systematic endoscopic evaluation and formal functional dyspepsia criteria means that undetected organic pathology—including gastroparesis, which is particularly relevant given the high diabetes prevalence (60.0%)—cannot be excluded. Helicobacter pylori status, physical activity, and psychological distress were also unmeasured. Collectively, these unmeasured confounders represent an important limitation that should be considered when interpreting the reported associations. Fourth, the high-adherence group comprised only 13 participants (7.9%), limiting the precision of category-based estimates; accordingly, the continuous regression analyses are considered the primary evidence. Fifth, no formal multiplicity correction was applied across the three SODA subscale outcomes, although these represent pre-specified, conceptually distinct dimensions. Finally, this study was conducted at a single tertiary-care geriatric outpatient clinic, which may limit generalizability.

## Conclusion

In conclusion, higher adherence to the Mediterranean diet was independently associated with lower dyspeptic symptom severity and greater symptom-related satisfaction among older adults with dyspepsia. These findings underscore the potential role of dietary quality as a modifiable factor in the management of dyspeptic symptoms in geriatric populations. If confirmed by prospective and interventional research, Mediterranean diet–based nutritional counseling could represent a valuable adjunct to conventional management strategies in geriatric care, particularly in the context of multimorbidity and polypharmacy. Future prospective and interventional studies are warranted to determine whether improving Mediterranean diet adherence can causally reduce dyspeptic symptoms and enhance quality of life in older adults.

## Supplementary Information

Supplementary Material 1: Supplementary Table S1. Sociodemographic and lifestyle factors associated with Mediterranean diet adherence categories.

Supplementary Material 2: Supplementary Table S2. Sensitivity analyses excluding the high-adherence group.

Supplementary Material 3: Supplementary File 1. Data collection form.

## Data Availability

The datasets generated and/or analyzed during the current study are not publicly available due to institutional data protection policies but are available from the corresponding author on reasonable request.
