# Supplementary material for: Association between Mediterranean diet adherence and dyspeptic symptoms in older adults: a cross-sectional study in a geriatric outpatient population
Source: BMC Geriatr. 2026 Apr 17;26:763. doi: 10.1186/s12877-026-07501-y (PMC13220539; doi:10.1186/s12877-026-07501-y)
Supplement: Supplementary file 1 — Supplementary Material 1: Supplementary Table S1. Sociodemographic and lifestyle factors associated with Mediterranean diet adherence categories. [file 12877_2026_7501_MOESM1_ESM.docx]

**Supplementary Table S1. Sociodemographic and lifestyle factors associated with Mediterranean diet adherence categories**

| **Variable** | **Low adherence (n=30)** | **Moderate adherence (n=122)** | **High adherence (n=13)** | **p value*** |
| --- | --- | --- | --- | --- |
| **Sex** |  |  |  |  |
| Female, n (%) | 24 (80.0) | 80 (65.6) | 13 (100.0) | **0.016** |
| Male, n (%) | 6 (20.0) | 42 (34.4) | 0 (0.0) |  |
| **Marital status** |  |  |  |  |
| Married, n (%) | 10 (33.3) | 89 (73.0) | 9 (69.2) | **<0.001** |
| Single, n (%) | 20 (66.7) | 33 (27.0) | 4 (30.8) |  |
| **Living arrangement** |  |  |  |  |
| Living alone, n (%) | 12 (40.0) | 18 (14.8) | 3 (23.1) | **0.008** |
| Living with family, n (%) | 18 (60.0) | 104 (85.2) | 10 (76.9) |  |
| **Meal-skipping behaviors** |  |  |  |  |
| Breakfast skipping (never),n(%) | 12 (40.0) | 68 (55.7) | 13 (100.0) | **0.004** |
| Lunch skipping (never),n(%) | 12 (40.0) | 50 (41.0) | 7 (53.8) | 0.255 |
| Dinner skipping (never),n(%) | 15 (50.0) | 80 (65.6) | 13 (100.0) | **0.010** |
| Snack skipping (never), n(%) | 10 (33.3) | 33 (27.0) | 5 (38.5) | **0.017** |

*Values are presented as number (percentage). Associations were assessed using the chi-square test or exact tests when appropriate. Mediterranean diet adherence was categorized as low, moderate, or high according to MEDAS scores.* *For meal-skipping variables, the category “never skipping” is presented. Variables with expected cell counts <5 were evaluated using exact methods.*
