# Supplementary material for: Association between Mediterranean diet adherence and dyspeptic symptoms in older adults: a cross-sectional study in a geriatric outpatient population
Source: BMC Geriatr. 2026 Apr 17;26:763. doi: 10.1186/s12877-026-07501-y (PMC13220539; doi:10.1186/s12877-026-07501-y)
Supplement: Supplementary file 2 — Supplementary Material 2: Supplementary Table S2. Sensitivity analyses excluding the high-adherence group. [file 12877_2026_7501_MOESM2_ESM.docx]

**Supplementary Table S2.** **Sensitivity analyses excluding the high-adherence group.**

|  | **Variable** | **B (95% CI)** | **Standardized β** | **p value** |
| --- | --- | --- | --- | --- |
| **Panel A**  **SODA**  **pain**  **intensity** | **Age (years)** | 0.24 (−0.01 to 0.49) | 0.151 | 0.056 |
|  | **Sex (female vs male)** | 9.08 (5.49 to 12.66) | 0.404 | **<0.001** |
|  | **BMI (kg/m²)** | −0.02 (−0.34 to 0.31) | −0.009 | 0.914 |
|  | **MEDAS score** | −0.63 (−1.76 to 0.50) | −0.084 | 0.275 |
|  |  |  |  |  |
|  | ***Model statistics:*** *R²=0.158, Adjusted R²=0.135, F=6.88, p<0.001* | | | |
| **Panel B**  **SODA**  **non-pain symptom scores** | **Variable** | **B (95% CI)** | **Standardized β** | **p value** |
|  | **Age (years)** | 0.11 (−0.02 to 0.24) | 0.124 | 0.093 |
|  | **Sex (female vs male)** | 3.82 (1.96 to 5.68) | 0.306 | **<0.001** |
|  | **BMI (kg/m²)** | −0.02 (−0.19 to 0.15) | −0.017 | 0.814 |
|  | **MEDAS score** | −1.87 (−2.46 to −1.28) | −0.450 | **<0.001** |
|  |  |  |  |  |
|  | ***Model statistics****: R²=0.267, Adjusted R²=0.247, F=13.40, p<0.001* | | | |
| **Panel C**  **SODA satisfaction score** | **Variable** | **B (95% CI)** | **Standardized β** | **p value** |
|  | **Age (years)** | 0.01 (−0.07 to 0.08) | 0.015 | 0.852 |
|  | **Sex (female vs male)** | −0.03 (−1.10 to 1.05) | −0.004 | 0.958 |
|  | **BMI (kg/m²)** | −0.11 (−0.20 to −0.01) | −0.168 | 0.035 |
|  | **MEDAS score** | 0.80 (0.46 to 1.14) | 0.354 | **<0.001** |
|  |  |  |  |  |
|  | ***Model statistics****: R²=0.169, Adjusted R²=0.146, F=7.46, p<0.001* | | | |

*The models were adjusted for age, sex, body mass index (BMI), and Mediterranean Diet Adherence Score (MEDAS), after exclusion of participants in the high-adherence group. No multicollinearity was detected (all VIF values <1.2).*
