# Supplementary material for: Association between Mediterranean diet adherence and dyspeptic symptoms in older adults: a cross-sectional study in a geriatric outpatient population
Source: BMC Geriatr. 2026 Apr 17;26:763. doi: 10.1186/s12877-026-07501-y (PMC13220539; doi:10.1186/s12877-026-07501-y)
Supplement: Supplementary file 3 — Supplementary Material 3: Supplementary File 1. Data collection form. [file 12877_2026_7501_MOESM3_ESM.docx]

**Supplementary File 3. Data Collection Form**

Questionnaire No: ……….
**1. Date of birth: ............................................................**
**2. Sex**
 1. Male 2. Female

**3. Marital status**
 1. Married 2. Single 3. Widowed 4. Divorced

**4. Educational status (please mark according to the highest level completed)**
 1. Illiterate-Literate
 2. Primary school
 3. Middle school- High school
 4. University or higher

**5. How would you describe your income–expense status?**
 1. Income is higher than expenses
 2. Income is lower than expenses
 3. Income is equal to expenses

**6. With whom do you live?**
 1. Alone
 2. With family
 3. With friends
 4. Other: ................................

**7. Height: .......... cm Weight: .......... kg**

**8. Do you have any physician-diagnosed disease that requires regular follow-up or ongoing treatment?**
 0. None
 1.Yes: .....................................................................................................

1. **9. Do you regularly use any medications?**
2. 1.No
3. 2.Yes → Please specify:…………………………………………………………….

   **10. How many meals do you eat per day?**
    **(.................. Main meals ...................... Snacks)**

   **11. Meal skipping status:**
   **Breakfast:** 1.Always / 2.Sometimes / 3.Never
   **Lunch:** 1.Always / 2.Sometimes / 3.Never
   **Dinner:** 1.Always / 2.Sometimes / 3.Never
   **Snacks:** 1.Always / 2.Sometimes / 3.Never
